# Determinants of repeated abortion among women of reproductive age attending health facilities in Northern Ethiopia: a case–control study

**DOI:** 10.1186/s12889-017-4106-1

**Published:** 2017-02-13

**Authors:** Mussie Alemayehu, Henock Yebyo, Araya Abrha Medhanyie, Alemayehu Bayray, Misganaw Fantahun, Gelila Kidane Goba

**Affiliations:** 1 School of Public Health, Mekelle University, College of Health Sciences, Mekelle, Ethiopia; 2 School of Public Health, Addis Ababa University, Addis Ababa, Ethiopia; 3 Department of Obstetrics and Gynecology, University of Illinois at Chicago, Chicago, USA

**Keywords:** Abortion, Single abortion, Repeated abortion, Tigray, Ethiopia

## Abstract

**Background:**

Every year, an estimated 19–20 million unsafe abortions take place, almost all in developing countries, leading to 68,000 deaths and millions more injured many permanently. Many women throughout the world, experience more than one abortion in their lifetimes. Repeat abortion is an indicator of the larger problem of unintended pregnancy. This study aimed to identify determinants of repeat abortion in Tigray Region, Ethiopia.

**Methods:**

Unmatched case–control study was conducted in hospitals in Tigray Region, northern Ethiopia, from November 2014 to June 2015. The sample included 105 cases and 204 controls, recruited from among women seeking abortion care at public hospitals. Clients having two or more abortions (“repeat abortion”) were taken as cases and those who had a total of one abortion were taken as controls (“single abortion”). Cases were selected consecutive based on proportional to size allocation while systematic sampling was employed for controls. Data were analyzed using SPSS version 20.0. Binary and multiple variable logistic regression analyses were calculated with 95% CI.

**Results:**

Mean age of cases was 24 years (±6.85) and 22 years (±6.25) for controls. 79.0% of cases had their sexual debut in less than 18 years of age compared to 57% of controls. 42.2% of controls and 23.8% of cases cited rape as the reason for having an abortion. Study participants who did not understand their fertility cycle and when they were most likely to conceive after menstruation (adjusted odds ratio [AOR] = 2.0, 95% confidence interval [CI]: 1.1–3.7), having a previous abortion using medication (AOR = 3.3, CI: 1.83, 6.11), having multiple sexual partners in the preceding 12 months (AOR = 4.4, CI: 2.39,8.45), perceiving that the abortion procedure is not painful (AOR = 2.3, CI: 1.31,4.26), initiating sexual intercourse before the age of 18 years (AOR = 2.7, CI: 1.49, 5.23) and disclosure to a third-party about terminating the pregnancy (AOR = 2.1, CI: 1.2,3.83) were independent predictors of repeat abortion.

**Conclusion:**

This study identified several factors correlated with women having repeat abortions. It may be helpful for the Government of Ethiopia to encourage women to delay sexual debut and decrease their number of sexual partners, including by promoting discussion within families about sexuality, to decrease the occurrence of repeated abortion.

## Background

Globally, unsafe abortion is the leading cause of death among pregnant women and causes 13% of all maternal deaths [1]. In developing countries, a woman dies every 8 min due to complications arising from unsafe abortion. An estimated 10–50% of women face life-threatening complications and long-term consequences such as incomplete abortion, infection and secondary infertility due to abortion [2–4]. Even among women for whom abortion is induced, approximately 10% experience immediate complications, of which one-fifth are considered major [5, 6]. Providing post-abortion service is a widely accepted public health strategy to reduce maternal morbidity and mortality. Linking abortion care and comprehensive family planning can help prevent future unwanted pregnancies and repeat abortions [7–9].

A study in Mozambique showed that most post-abortion clients (86%) had a prior pregnancy and almost half (44%) an abortion before the current one [10]. Repeat abortion is associated with many problems, including poor health [11]. Women who had one, two or more previous induced abortions are 1.89, 2.66 and 2.03 times more likely, respectively, to have a subsequent pre-term delivery, compared to women who carry to term. Prior induced abortion not only increases the risk of premature but also delayed delivery [12]. The risk of repeat abortion within the abortion population follows a bell-curve: 7.3% are adolescents, 30.8% are 30–34 years old and 13.0% are above the age of 44 [13].

The magnitude of repeat abortion in Ethiopia is not known, which this study seeks to help illuminate. The overall prevalence of unintended pregnancy in Ethiopia is about 42%. Out of an estimated 3.27 million pregnancies annually, 500,000 ends up in either spontaneous or unsafely induced abortion [8]. This study can help policymakers working in the area of maternal health to promote the well-being of mothers and women of reproductive age by identifying determinants of repeat abortion by testing the hypothesis “there was a difference in factors that determine for having abortion among women of reproductive age group in those with once abortion and having repeated abortion in terms of contraceptive use, client factors and health service related factors”.

## Methods

An unmatched case–control study was conducted in hospitals in Tigray Region, northern Ethiopia, from November 2014 to June 2015. The sample size was calculated by considering the proportion of contraceptive non-use among first-time (48.9%) and repeat (30.9%) abortion seekers as shown in other studies in Ethiopia [14]. It was calculated using Epi-info version 7. We used 95% CI and 80% power of the test with a 1:2 ratio of cases to controls. A total sample size of 315 was determined, including105 cases and 210 controls, which also accounted for 10% non-response.

There are 16 governmental and two private hospitals as well as 256 health centers in Tigray [15]. From these 16 governmental hospitals, we randomly selected eight hospitals. In these hospitals, all clients 15–49 years of age who came for seeking post-abortion care for at least the second time were considered as cases (“repeat abortion”). Those seeking care following their first abortion were considered as controls (“single abortion”). First, the total sample size of both case and controls was proportionally allocated to the selected hospital based on their 3-month previous caseload. Then, to select controls, we used systematic sampling technique, but for cases, we enrolled all women (consecutive) until the required sample size was filled.

Data were collected using a structured questionnaire; administered in-person by 12 nurse interviewers experienced in abortion care and 4 health officers was deployed for supervision. Questionnaires included socio-demographic and economic variables, health and healthcare-related factors and knowledge about contraceptive use. Questionnaires were adapted from different literature and took into account the local context [16–18]. History of abortion either single or repeat abortion was identified from medical records of the patients. Besides, so as to minimize bias the patient was asked either the patient comes to seek abortion service for the first time or second and above. And the patient response was checked with the medical record. And in the case of inconsistent finding, the patient response was taken. The outcome variable of this study was an episode of abortion either single or repeat (two and more). Furthermore, in this study, “surgical abortion” includes abortion from manual vacuum aspiration (MVA) or curettage.

The questionnaire was prepared in English and translated to Tigrigna. It was checked for consistency by back-translation to English by two different individuals. The data collection process was strictly supervised and data was checked for consistency and completeness. Incomplete and unclear questionnaires were returned to interviewers to be completed.

### Data analysis

Data were entered, cleaned and analyzed using SPSS 20 for Windows (SPSS Inc. Version 20, Chicago, Illinois). Data cleaning was done by running frequencies, cross-tabulation and sorting among reported cases or variables. Frequency and mean were obtained for variables. A “binary analysis” was used to describe the association between independent and dependent variables and a multiple variable logistic regression analysis was used to show factors determining outcome variables. Before proceeding to the multivariable logistic regression, variables which had a *p*-value of 0.25 or less in the binary logistic regression were included in the multivariable logistic regression. Finally, *P*-value < 0.05 was considered statistically significant for all independent variables at the multivariable logistic regression.

Crude odds ratio (COR) and adjusted odds ratio (AOR) were calculated. To determine the factors most statistically significantly associated with repeat abortion, odds ratio at 95% CI was determined using logistic regression analysis. The final model was fitted using the Hosmer–Lemeshow Goodness of Fit Test and multicollinearity were checked to minimize bias. The goodness of fit of the final model was checked by using the Hosmer–Lemeshow Goodness of Fit Test and *p*-value greater than 0.05 considered as the model fit to the logistic regression. For the multicollinearity, we use Variance Inflator Factor (VIF) to the inquiry instability of the effect size of predictors as the result of high collinearity among themselves. The multicollinearity was checked by using mean, variance inflation factors (VIF) cutoff point of 10.

## Results

### Socio-demographic characteristics of study participants

Of the 314 women who visited abortion clinics at selected hospitals during the study period, 309 completed the interview, a response rate of 98.4%. And the main reason for non-response was a refusal to participate in to study. Among these, 105 (33.9%) were cases (“repeat abortion”) and 204 (66.1%) controls (“single abortion”). The mean age of cases was 24 years (Min = 16, Max = 42, SD = 6.85) and 22 years (Min = 15, Max = 42, SD = 6.25) for controls. The majority of subjects were from urban areas: 65.7% of cases and 75.5% for controls. Orthodox Christian and Tigrawot were the predominant religious and ethnic groups, respectively: 78.1% and 83.8% of cases were Orthodox Christian and Tigrawot, respectively, compared to 88.2% and 87.3%, respectively, of controls. Nearly one-fourth (24.8%) of cases and18.6% of controls were unable to read and write. Just over 30% of cases had attended grades 9–12 compared to 37.6% of controls. Roughly half of cases and controls were married. Over 40% of controls were students and 35% of cases were employed. Approximately 60% of controls and cases did not have their own incomes (Table 1).

**Table 1:** Socio demographic characteristics of the study respondents, Tigray region, 2015

Variables	Controls	Cases	Total	*P*-Value
	*N* (%)	*N* (%)	*N* (%)	
Age				
Less than 18	38 (18.6)	10 (9.5)	48 (15.5)	0.15
18 and above	166 (81.4)	95 (90.5)	261 (84.5)	
Residence				
Urban	154 (75.5)	69 (65.7)	223 (72.2)	0.049
Rural	50 (24.5)	36 (34.3)	86 (27.8)	
Religion				
Orthodox	180 (88.2)	82 (78.1)	262 (84.7)	0.006
Other^a^	24 (11.8)	23 (21.9)	47 (15.3)	
Ethnicity				
Tigrawot	178 (87.3)	88 (83.8)	266 (86.1)	0.39
Other^b^	26 (12.7)	17 (16.2)	43 (13.9)	
Educational status				
Unable to read and write	38 (18.6)	26 (24.8)	64 (20.7)	0.41
Able to read and write	21 (10.3)	10 (9.5)	31 (10.0)	
1–8 grade	35 (17.2)	22 (21.0)	47 (15.2)	
9–12 grade	77 (37.7)	33 (31.4)	110 (35.5)	
College or university level	33 (16.2)	14 (13.3)	47 (15.2)	
Marital status				
Married	81 (39.7)	55 (52.4)	136 (44)	0.10
Single	97 (47.5)	38 (36.2)	135 (43.7)	
Divorced	21 (10.3)	12 (11.4)	33 (10.7)	
Widowed	5 (2.5)	-	5 (1.6)	
Occupation				
Students	87 (42.6)	34 (32.4)	121 (39.2)	0.10
Housewives	50 (24.5)	34 (32.4)	84 (27.2)	
Employed	67 (32.8)	37 (35.2)	104 (33.7)	
Had own income				
Yes	74 (36.3)	43 (41.0)	117 (37.9)	0.53
No	130 (63.7)	62 (59.0)	192 (62.1)	

^a^Muslim and protestant

^b^Amhara and Oromo

### Reproductive history of study participants

Seventy-nine percent (79.0%) of cases and 57.4% of controls had their first sexual intercourse before the age of 18 years. More than four-fifths (86.3%) of controls had ever been pregnant previously. In terms of sexual partners, 46.6% of controls and 72.4% of cases had two or more sexual partners ever and 19.1% of cases and 41% of controls had two or more partners in the 12 months preceding the survey, respectively. As such, cases were approximately twice as likely to have had two or more sexual partners. Nearly 80% of controls and 67.6% of cases want to have children in the future (Table 2).

**Table 2:** Reproductive history of the study respondents, Tigray region, 2015

Variables	Controls	Cases	Total	*P*-Value
	*N* (%)	*N* (%)	*N* (%)	
Age at first sexual intercourse				
Less than 18	117 (57.4)	83 (79.0)	200 (64.7)	<0.01
18 and above	87 (42.6)	22 (21.0)	109 (35.9)	
Ever number of sexual partners				
One	109 (53.4)	29 (27.6)	138 (44.7)	<0.01
Two and above	95 (46.6)	76 (72.4)	171 (55.3)	
Number of sexual partners in the past 12 months				
One	165 (80.9)	62 (59.0)	227 (73.5)	<0.01
Two and above	39 (19.1)	43 (41.0)	82 (26.5)	
Ever been pregnant before				
Yes	176 (86.3)	105 (100.0)	281 (90.9)	<0.01
No	28 (13.7)	-	28 (9.1)	
Did want to have children for the future				
Yes	161 (78.9)	71 (67.6)	232 (75.1)	0.05
No	43 (21.1)	34 (32.4)	77 (24.9)	

### Abortion-related characteristics of respondents

Rape was the reason 42.2% of controls and 23.8% cases sought an abortion. A further 14.2% of controls and 13.3% of cases cited incest as the reason for seeking an abortion. In terms of a number of abortions, 87.6% of cases had two previous abortions while10.5% and 1.9% had three and four or more previous abortions, respectively. Most (78.4%) controls and 52.4% of cases reported that the current abortion was done surgically. Over half, (54.9%) of controls and 30.5% of cases indicated the abortion procedure was painful.

Fourteen percent (14.2%) and 22.1% of controls and 13.3% and 18.1% of cases did not receive counseling about family planning or instruction on when to return to fertility after the abortion, respectively. A significant number of women had some complication while they were pregnant, including 21.1% of controls and 29.5% of cases. Bleeding was the most common complication and was experienced by 74.4% of controls and 80.6% of cases. Approximately, 30% of women in both groups faced psychological problems after the procedure.

Forty-four percent (44.1%) of controls and 58.1% of cases disclosed to another individual about terminating the pregnancy. Majority (51.1%) of controls and 44.3% of cases disclosed to their friends (Table 3).

**Table 3:** Abortion related characteristics of the respondents, Tigray region, 2015

Variables	Controls	Cases	Total	*P*-Value
	*N* (%)	*N* (%)	*N* (%)	
Reason for abortion				
Family planning is not effective	12 (5.9)	18 (17.1)	30 (9.9)	<0.01
Unplanned sex	41 (20.1)	16 (15.2)	57 (18.4)	
Rape	86 (42.2)	25 (23.8)	111 (35.9)	
Pregnancy from family	29 (14.2)	14 (13.3)	43 (13.9)	
Others	36 (17.6)	32 (30.5)	68 (22.0)	
Abortion was done using				
Medical abortion	44 (21.6)	50 (47.6)	94 (30.4)	<0.01
MVA or curettage	160 (78.4)	55 (52.4)	215 (69.6)	
Abortion procedure was painful				
Yes	112 (54.9)	32 (30.5)	144 (46.6)	<0.01
No	92 (45.1)	73 (69.5)	165 (53.4)	
Did you get counseling about FP				
Yes	175 (85.8)	91 (86.7)	266 (86.1)	0.08
No	29 (14.2)	14 (13.3)	43 (13.9)	
Did you get counseling when fertility is return after the procedure				
Yes	159 (77.9)	86 (81.9)	245 (79.3)	0.20
No	45 (22.1)	19 (18.1)	64 (23.0)	
When it return fertility				
Within 2 weeks	74 (46.5)	32 (37.2)	106 (43.3)	0.24
Two weeks and above	85 (53.5)	54 (62.8)	139 (56.7)	
Did you face complication while you were pregnant				
Yes	43 (21.1)	31 (29.5)	74 (23.9)	0.24
No	161 (78.9)	74 (70.5)	235 (76.1)	
Type of complication				
Bleeding	32 (74.4)	25 (80.6)	57 (77)	0.50
Mechanical trauma	5 (11.6)	5 (16.1)	10 (13.5)	
Infection	6 (14)	1 (3.2)	7 (9.5)	
Did you face psychological problem				
Yes	56 (27.5)	30 (28.6)	86 (27.8)	0.73
No	148 (72.5)	75 (71.4)	223 (72.2)	
Did you disclosure to any one				
Yes	90 (44.1)	61 (58.1)	151 (48.9)	0.02
No	114 (55.9)	44 (41.9)	158 (51.1)	
To whom did you disclose				
Partner	19 (21.1)	19 (31.1)	38 (25.2)	0.45
Family	25 (27.8)	15 (24.6)	40 (26.5)	
Friend	46 (51.1)	27 (44.3)	73 (48.3)	

### Health-related characteristics of respondents

Two-thirds of controls and cases had ever used family planning. Of these, 59.4% of controls and 72.2% of cases had ever used injectable contraceptives. Emergency contraceptives were ever used by 38.3% of controls and 43.3% of cases. Among women who took an emergency contraceptive, a considerable percentage—22.7% of controls and 50% of cases –had ever taken emergency contraceptives within 1 day of unprotected sexual intercourse. More than six in ten cases and controls thought that their menstrual flow was regular. Three-fourths of controls and 67.6% of cases thought that repeat abortion results in infertility (Table 4).

**Table 4:** Health related characteristics of the respondents, Tigray region, 2015

Variables	Controls	Cases	Total	*P*-Value
	*N* (%)	*N* (%)	*N* (%)	
Have you ever use FP				
Yes	138 (67.6)	72 (68.6)	210 (68.2)	0.86
No	66 (32.4)	33 (31.4)	99 (32.0)	
Type of FP				
Injectable	82 (59.4)	52 (72.2)	134 (63.8)	0.13
Pill	41 (29.7)	10 (13.9)	51 (24.3)	
Implanon	9 (6.5)	4 (5.6)	13 (6.2)	
IUCD	1 (0.7)	-	1 (0.5)	
condom	5 (3.6)	6 (8.3)	11 (5.2)	
Have you ever use emergency contraceptive (EC)				
Yes	44 (38.3)	26 (43.3)	70 (40.0)	0.51
No	71 (61.7)	34 (56.7)	105 (60.0)	
After what time did you use EC				
Within 1 day	10 (22.7)	13 (50)	23 (32.9)	0.08
After 1 day	14 (31.8)	2 (7.7)	16 (22.9)	
After 2 day	15 (34.1)	3 (11.5)	18 (25.7)	
After 3 day	5 (11.4)	8 (30.8)	13 (18.6)	
Did you have plan to use FP for the future				
Yes	184 (90.2)	89 (84.8)	273 (88.3)	0.18
No	20 (9.8)	16 (15.2)	36 (11.7)	
Did you know when the fertility is return after manustration				
Yes	140 (68.6)	56 (53.3)	196 (63.4)	0.01
No	64 (31.4)	49 (46.7)	113 (36.6)	
When to return fertility after manustration				
1–8 days	45 (32.1)	6 (10.7)	51 (26)	0.01
9–18 days	84 (60.0)	46 (82.1)	130 (66.3)	
After 18 days	11 (7.9)	4 (7.1)	15 (7.7)	
Regularity of the manustration				
Regular	129 (63.2)	63 (60.0)	192 (62.1)	0.69
Irregular	67 (32.8)	38 (36.2)	105 (34.0)	
I do no	8 (3.9)	4 (3.8)	12 (3.9)	
Having a repeated abortion can result in abortion				
Agree	129 (63.2)	63 (60)	192 (62.1)	0.04
I do no	67 (32.8)	38 (36.2)	105 (34.0)	
Disagree	8 (3.9)	4 (3.8)	12 (39.0)	

### Predictors of repeated abortion

Adjusting for other variables, the odds of repeat abortion was 2.0 times with (AOR = 2.0, CI: 1.12, 3.69) more likely with women who did not understand their fertility cycles and when they were most likely to conceive after menstruation as compared with their counterparts. And the odds of repeat abortion was 3.3 times with (AOR = 3.3, CI: 1.83, 6.11) more likely among women who previously used medication for an abortion procedure as compared women who had a surgical abortion procedure. The odds of repeat abortion was 4.4 times with (AOR = 4.4, CI: 2.39, 8.45) more likely among women who had multiple sexual partners in the past 12 months as compared to those who had one sexual partner. Besides, the odds of repeat abortion was 2.3 times with (AOR = 2.3, CI: 1.31, 4.26) more likely among women who perceived that the abortion procedure was not painful as compared to those who thought that the procedure was painful. Furthermore, the odds of repeat abortion was 2.7 and 2.1 times with (AOR = 2.7, CI = 1.49, 5.23) and (AOR = 2.1, CI: 1.23, 3.83) more likely among women who started their first sexual intercourse before 18 years and women that disclosed to a third-party about having an abortion as compared to their counterparts, respectively (Table 5).

**Table 5:** Predictors of repeated abortion, Tigray region, 2015

Variables	Abortion		COR [95%]	AOR [95%]
	Repeated	Single		
	*N* (%)	*N* (%)		
Know when to return fertility after menstruation				
Yes	56 (53.3)	140 (68.6)	1	1
No	49 (46.7)	64 (31.4)	1.9 (1.17,3.1)	2.0 (1.12,3.69)
Abortion done				
Medical abortion	50 (47.6)	44 (21.6)	3.3 (1.98,5.49)	3.3 (1.83,6.11)
Surgical abortion	55 (52.4)	160 (78.4)	1	1
Perceive that the abortion procedure was painful				
Yes	32 (30.5)	112 (54.9)	1	1
No	73 (69.5)	92 (45.1)	2.7 (1.68,4.57)	2.3 (1.31,4.26)
Age at first sexual intercourse				
Less than 18	83 (79)	117 (57.4)	2.8 (1.62,4.84)	2.7 (1.49,5.23)
18 and above	22 (21)	87 (42.6)	1	1
Sexual partner in the past 12 months				
One	29 (27.6)	109 (53.4)	1	1
Two and above	76 (72.4)	95 (46.6)	2.9 (1.74,4.94)	4.4 (2.39,8.45)
Abortion disclosure				
Yes	61 (58.1)	90 (44.1)	1.7 (1.09,2.82)	2.1 (1.23,3.83)
No	44 (41.9)	114 (55.9)	1	1
Repeated abortion results in sterility				
Agree	63 (60)	129 (63.2)	0.2 (0.09,0.78)	0.3 (0.1,12)
I do no	38 (36.2)	67 (32.8)	0.3 (0.1,1.02)	0.4 (0.11,1.45)
Disagree	4 (3.8)	8 (3.9)	1	1

## Discussion

Adjusting for other variables, study participants who did not understand their fertility cycle and when they were most likely to conceive after menstruation, having an abortion procedure previously with medication abortion, having multiple sexual partners in the past 12 months, perceiving that the abortion procedure was not painful, initiating sexual intercourse before the age of 18 years and disclosing about terminating the pregnancy to a third-party were independent predictors of repeat abortion.

Even though the mean age distribution of study participants was relatively similar, there were differences in socio-demographic characteristics. Controls were slightly more educated. Cases were more likely to be housewives (32.4%) and controls more likely to be students (42.6%). Seventy-nine percent (79%) of cases had their sexual debut at less than 18 years of age compared to 57% of controls. Cases were also approximately twice as likely to have had multiple sexual partners.

Another study suggests that the prevalence of repeated abortion is high in Ethiopia and that most women seeking repeat abortion do so to financial reasons as well as the desire to stop having children [19]. Our study revealed that rape, unplanned sex and incest were among the leading reasons women sought abortions. The National Technical and Procedural Guideline for Safe Abortion permits termination of pregnancy in cases of pregnancy resulting from incest or rape when continuation of the pregnancy endangers the life of the mother, the fetus has an incurable and serious deformity, and the pregnant woman has a physical or mental deficiency [8]. It’s possible that women are increasingly aware of the laws governing abortion in Ethiopia, particularly as related to rape and incest, and that they are choosing to have procedures at public facilities at little or no cost.

A study in Addis Ababa found that 69.1% of post-abortion patients had ever used contraceptives and majority of these ever used injectables [18]. This is consistent with the findings in our study. More than two-thirds of cases and controls had ever used contraceptives and injectable contraceptive was the most common contraceptive ever used. A study of post-abortion care (PAC) in public health facilities of Ethiopia indicated that only two in ten women asked for contraceptives during post-abortion care (PAC) visits and providers offered information on contraception to approximately half of all women [20].

Our study showed that more than 80% of cases and controls intend to use family planning in the future, although one-third does not know when their fertility resumes after having an abortion. A study in Kano, Nigeria, showed that in 13% of observed cases, the provider did not explain that the patient had an immediate risk of repeat pregnancy if she did not use contraception following an abortion [21]. This may indicate that Ethiopia is missing an important opportunity to provide contraceptive to women who are likely to need family planning. PAC providers should offer information about family planning, including side-effects, method mix and effective period, to help PAC patients avoid future abortions. A study in Bolivia showed 97% of PAC patients were counseled about family planning after improved PAC services were introduced [22], which could be a model for a similar initiative in Ethiopia.

Different studies show that abortion increase the risk of complications, namely: placenta previa, which increases the risk of fetal malformation, perinatal death and excessive bleeding during labor [23]. Multiple abortions have been found to correlate with poor health [11]. This is somewhat in line with our findings. Our study showed that a considerable percentage of cases had complications while they were pregnant and that most complications were from bleeding, mechanical-related issues, trauma and infection.

For most couples, an abortion causes unforeseen problems in the relationship. Post-abortion couples are more likely to divorce or separate. After an abortion, many women develop a greater difficulty forming lasting bonds with a male partner [24]. In our study, approximately three out of ten women had psychological problems associated with the abortion, including lowered self-esteem, greater distrust of men and sexual dysfunction. However, marital status was not a significant determinant of repeat abortion in our study.

Eight of ten women in our study initiated sexual intercourse before the age of 18 years. Different studies show there’s a strong correlation between the earlier sexual debut and abortion [25]. In our study, women who started sexual intercourse before the age of 18 had odds three times higher of being a case than those who started at 18 or later. The Government should work to help adolescents delay sexual debut as well as encouraging family planning, including by empowering communities and particularly women, to freely discuss sexuality with young girls at home.

The number of sexual partners a woman has also tied to higher rates of repeat abortion. Studies show that 40 – 50% of women who have had 10 or more (male) lifetime sexual partners have had an abortion [26] and almost 90% of women who have had at least one abortion have had three or more sexual partners [27]. This is in line with our findings, which showed the odds of repeated abortion were four times higher among women who had two or more sexual partners as compared with those who had one sexual partner. The Government should continue to encourage women to limit and reduce their number of sexual partners, both as a way to reduce HIV and STI transmission, but also as a means of preventing abortion.

Our study showed women who used medication for the current abortion were more likely to have had repeated abortions than those who had a surgical abortion. Given that medical abortion seems natural, with no shots, anesthesia, instruments or vacuum aspirator, and allows the woman to be at home instead of in a clinic, this method of abortion may seem more comforting, private and low-risk to women [28, 29]. Even though medical abortion has several advantages, it fails more often than surgical abortion, requires at least two visits, and entails longer cramping and bleeding than occurs after a surgical abortion.

The odds of repeated abortion among women who disclosed to a third-party about terminating the pregnancy were two times as high as those women that did not disclose to a third-party. This implies that disclosing the procedure may provide women the psychological or financial support needed to carry out the procedure.

Even though, our study tried to identify determinant factors of repeat abortion among women seeking abortion care, it was limited by the fact that identifying women who had previous abortions in other health facilities were challenging due to the sensitivity of the subject, although our study endeavored to take a detailed history whether the woman had repeat abortions or not. Moreover, although rape and incest combined were identified by 40% of cases and 55% of controls as the reason for seeking an abortion, we were not able to validate these seemingly high rates through external sources like police records. Given that the law in Ethiopia permits abortion in the case of rape and incest, it is worth exploring to see if these high rates of reported rape and incest represent the complete picture. Finally, selecting cases consecutively may not give equal chance for the women to be enrolled in our study.

## Conclusions

This study identified several factors correlated with women having repeat abortions in Tigray, Ethiopia. It may be helpful if the Government of Ethiopia encourages women to delay sexual debut and decrease their number of sexual partners, including by promoting discussion within families about sexuality, to decrease the occurrence of repeated abortion.

## Abbreviation

AOR: Adjusted odd ratio
CI: Confidence interval
COR: Crude odd ratio
EDHS: Ethiopian demographic health survey
Max: Maximum
MIN: Minimum
MVA: Manual vacuum aspiration
PAC: Post abortion care
SD: Standard deviation
SPSS: Statistical package for social sciences
VIF: Variance inflation factor
